# Role of prolyl hydroxylase domain proteins in the regulation of insulin secretion

**DOI:** 10.14814/phy2.12722

**Published:** 2016-03-20

**Authors:** Mei Huang, Sabina Paglialunga, Julia M.‐K. Wong, Monica Hoang, Renjitha Pillai, Jamie W. Joseph

**Affiliations:** ^1^ School of PharmacyUniversity of WaterlooWaterlooOntarioCanada

**Keywords:** *α*‐ketoglutarate, ethyl‐3,4‐dihydroxybenzoate, insulin release, islets, metabolism, pancreas, prolyl hydroxylase

## Abstract

Type 2 diabetes is associated with impaired nutrient‐regulated anaplerosis and insulin secretion in pancreatic *β*‐cells. One key anaplerotic substrate that may be involved in regulating insulin release is *α*‐ketoglutarate (*α*KG). Since prolyl hydroxylase domain proteins (PHDs) can metabolize cytosolic *α*KG, we sought to explore the role of this enzyme in the regulation of *β*‐cell function. The oxygen‐sensing PHDs regulate the stability of hypoxia‐inducible factor 1*α* (HIF1*α*) as well as other proline‐containing proteins by catalyzing the hydroxylation of proline residues. This reaction is dependent on sufficient levels of oxygen, iron, and *α*KG. In the present study, we utilized both pharmacological and genetic approaches to assess the impact of inhibiting PHD activity on *β*‐cell function. We demonstrate that ethyl‐3,4‐dihydroxybenzoate (EDHB), a PHD inhibitor, significantly blunted glucose‐stimulated insulin secretion (GSIS) from 832/13 clonal cells, rat, and human islets. EDHB reduced glucose utilization, ATP/ADP ratio, and key TCA cycle intermediates such as pyruvate, citrate, fumarate, and malate. siRNA‐mediated knockdown of PHD1 and PHD3 inhibited GSIS, whereas siRNA‐mediated knockdown of PHD2 had no effect on GSIS. Taken together, the current results demonstrate an important role for PHDs as mediators of islet insulin secretion.

## Introduction

Type 2 diabetes is associated with whole‐body insulin resistance and reduced pancreatic *β*‐cell insulin secretion. Before addressing the causal factors responsible for altered insulin secretion seen during type 2 diabetes, it is imperative to understand the regulation of *β*‐cell insulin secretion under normal physiological conditions. In *β*‐cells, glucose is taken up by the glucose transporter 2 (GLUT2) and is metabolized by glycolysis to produce pyruvate. Pyruvate enters the tricarboxylic acid cycle (TCA), generating reducing equivalents (NADH and FADH_2_), which ultimately results in an increase in ATP production through oxidative phosphorylation. The rise in ATP/ADP ratio in the cytosol closes plasma membrane ATP‐sensitive potassium channel (K_ATP_) leading to membrane depolarization and opening of voltage‐gated calcium channel (Wollheim and Sharp 1981; Rorsman 1997; Ashcroft and Gribble 1999). Calcium influx stimulates insulin granule exocytosis (Henquin et al. 2003). This pathway is commonly referred to as the K_ATP_ channel‐dependent or triggering pathway.

Another pathway that is thought to be important in regulating insulin secretion is called the K_ATP_ channel‐independent pathway or the amplifying pathway. This amplification pathway may generate signals such as a rise in the NADPH/NADP^+^ ratio and cytosolic *α*‐ketoglutarate (*α*KG) that can increase glucose‐stimulated insulin secretion (GSIS) (Odegaard et al. 2010; Huypens et al. 2011, 2012). Previously, we have shown that aryl hydrocarbon receptor nuclear translocator/hypoxia‐inducible factor‐1*β* (ARNT/HIF1*β*) is a key regulator of *β*‐cell function, in part, because it can regulate anaplerosis and the K_ATP_ channel‐independent pathway of insulin secretion (Pillai et al. 2011).

Hypoxia‐inducible factor 1*α* and ARNT/HIF1*β* are transcription factors involved in the cellular response to hypoxia. The response to hypoxia is highly regulated and under normoxic conditions, prolyl hydroxylase domain (PHD) enzymes direct HIF1*α* toward the ubiquitination and degradation pathway. PHDs are members of *α*‐KG‐dependent dioxygenase superfamily. These iron‐ and oxygen‐sensitive enzymes can initiate ubiquitination and proteolysis of proteins by hydroxylating proline residues (Myllyharju and Koivunen 2013). Once hydroxylated, these proteins bind to von Hippel–Lindau (VHL) tumor suppressor protein followed by ubiquitination by the E3 ubiquitin ligase complex and then targeted for rapid proteasomal degradation (Maxwell et al. 1999).

The *α*‐KG‐dependent dioxygenase superfamily of enzymes catalyze a diversity of reactions, in which decarboxylation of *α*‐KG produces succinate and CO_2_ and an active oxygen species leading to the hydroxylation of the primary substrate (Myllyharju 2013). There are three mammalian isoforms of PHDs (PHD1‐3), also known as EGLN proteins, and have distinct tissue expression profiles (Lieb et al. 2002) and subcellular localization (Metzen et al. 2003). PHD knockout mice have been widely studied to examine hypoxic mechanisms (Takeda and Fong 2007; Aragones et al. 2008; Adluri et al. 2011; Rishi et al. 2015). While, PHD1 and PHD3 knockout mice are viable, PHD2‐deficiency in mice leads to embryonic death due to placental defects (Takeda et al. 2006). PHD2 knockout in adipocytes results in mice resistant to diet‐induced obesity (Matsuura et al. 2013), whereas liver‐specific PHD3‐ablation improves insulin sensitivity and ameliorates diabetes (Taniguchi et al. 2013). These studies suggest that PHDs may play an important role in glucose homeostasis.

Prolyl hydroxylase domain metabolism of *α*‐KG could play a key role in regulating insulin secretion. Support for this comes from studies showing that *α*‐KG can act as an insulin secretagogue (Rabaglia et al. 2005) and inhibition of *α*‐KG‐dependent hydroxylases blocks GSIS (Fallon and MacDonald 2008; Cheng et al. 2010). These studies suggest iron/*α*‐KG‐dependent hydroxylases might be important effectors in defining the extramitochondrial role of *α*‐KG in insulin secretion. Here, we show that EDHB (ethyl‐3,4‐dihydroxybenzoate), a potent PHD inhibitor (Wang et al. 2002; Li et al. 2008), suppressed insulin secretion from clonal *β*‐cells and primary islets. EDHB also effectively blunted the glucose‐stimulated increase in the ATP:ADP ratio and the increase in TCA intermediates. These effects were primarily mediated through PHD3 inhibition as targeted siRNA‐mediated knockdown of PHD3 resulted in similar inhibition of GSIS.

## Methods

### Reagents

All reagents were obtained from Sigma (St. Louis, MO) unless otherwise specified.

### Cell lines

The 832/13 cell line (Hohmeier et al. 2000), derived from INS‐1 rat insulinoma cells (Asfari et al. 1992), was used for these experiments. The cells were a gift from C. B. Newgard and were cultured as described previously (Hohmeier et al. 2000; Huypens et al. 2011; Pillai et al. 2011; Patterson et al. 2014). Cell viability was determined using a commercially available kit and normalized compared to control no treatment (CellTiter‐Blue; Promega, Madison, WI).

### Islet isolation

Human islets were provided by the Alberta Diabetes Institute Islet Core at the University of Alberta in Edmonton, with the assistance of the Human Organ Procurement and Exchange Program and the Trillium Gift of Life Network in the procurement of donor pancreata for research (islets from four donors with an average age of 60) (Korbutt et al. 1996; Kin and Shapiro 2010). Rat islets were isolated and cultured as described previously (Huypens et al. 2011; Pillai et al. 2011). Experiments involving animals were approved by the local ethics committee.

### Glucose utilization and oxygen consumption

Glucose utilization was measured in 832/13 cells using [5‐H^3^]‐glucose (0.08 μCi/*µ*mol) and the samples were processed as described elsewhere (Huypens et al. 2011). Oxygen consumption was measured using a XF24 extracellular flux analyzer (Seahorse Bioscience, Billerica, MA) in 5 × 10^4^ cells/well as reported previously (Patterson et al. 2014). Briefly, oxygen consumption was measured in response to 2 mmol/L glucose, 10 mmol/L glucose ± EDHB (at concentrations indicated in the figure legends), 5 μmol/L oligomycin (Oligo), 50 μmol/L 2,4‐dinitrophenol (DNP) with 20 mmol/L pyruvate (Pyr), and 5 μmol/L rotenone (Rot) and 5 μmol/L myxothiazol (Myx).

### Nucleotide measurements

Cell nucleotide levels were measured using HPLC as described previously (Patterson et al. 2014). Briefly, 832/13 cells were plated in a six‐well plate. After 72 h, cells were preincubated for 2 h with KRB containing 2 mmol/L glucose at 37°C, 5% CO_2_. Cells were then treated with KRB containing either 2 mmol/L or 10 mmol/L glucose for 1 h. Two wells of the six‐well plate were pooled and nucleotides were extracted and then quantified. ATP, ADP, NADP^+^, and NADPH levels were separated by HPLC and then detected using a UV detector.

### TCA cycle intermediate measurements

After the insulin secretion assay, cells were washed once with ice‐cold PBS, scraped off the plates followed by centrifugation at 1200 g for 1 min at 4°C. The supernatant was discarded and the cell pellet was resuspended in 100 μL of water followed by sonicating for 60 sec (~40 kHz, ~140 W). Methanol (1 mL) was added followed by centrifugation for 5 min at 17,000 g. Supernatant was mixed with 5 μL of 0.25 mg/mL myristic acid‐d_27_ as an internal standard (IS; used for retention time locking) followed by drying with nitrogen. Samples were then derivatized and assayed for TCA cycle intermediates as described previously (Huang and Joseph 2014).

### Insulin secretion assay

Insulin secretion in response to glucose was measured as described previously for 832/13 cells and islets (Hohmeier et al. 2000; Huypens et al. 2011; Pillai et al. 2011; Patterson et al. 2014). After the assay, the buffer was collected and assayed for insulin using a Coat‐A‐Count radioimmunoassay kit (Siemens, Los Angeles, CA).

### siRNA transfection against PHD

Expression of PHD was suppressed in 832/13 cells by the introduction of siRNA duplexes (Integrated DNA Technologies, Coralville, IA). Three independent siRNA sequences were designed per PHD isoform (see Table 1). A previously used scrambled siRNA sequence (GAG ACC CUA UCC GUG AUU A) with no known target was used as a control (siCtl) (Pillai et al. 2011). siRNA duplexes were introduced into 832/13 cells at 40–50% confluence using INTERFERin nucleofection (Polyplus Transfection, New York, NY). RNA isolation and real‐time PCR was performed as described earlier (Patterson et al. 2014).

**Table 1:** siRNA duplex sequences. Three siRNA duplexes were constructed against each target gene. Relative to the start codon, the 5′ end of the siRNA target sequence corresponded to the following nucleotide

Target and accession no.	Duplex name	5′ no.	Sequence
PHD1 (EGLN2)	PHD1_1	1220	5′‐GGG AUU GUC AAC AUG CCU CAC GUA CCC‐3′
	PHD1_2	359	5′‐UGA CAC AGA CCC UGG CAA CUG AGG GAG‐3′
XM_006228575.2	PHD1_3	823	5′‐GCU UCC UCC CUC AGC CUA GAA CUU GCC‐3′
PHD2 (EGLN1)	PHD2_1	1232	5′‐UCU GCG UGG UGG ACG ACU UCC UGG G‐3′
	PHD2_2	1726	5′‐GUA UGC AGG CUG UAC UUC AUG AGG GUU‐3′
XM_008772679.1	PHD2_3	1325	5′‐AGU CAC UCU UCU GGC UGA CCA GUU GCC‐3′
PHD3 (EGLN3)	PHD3_1	1166	5′‐CCU UUC UUC AGC AUC GAA GUA CCA GAC‐3′
	PHD3_2	983	5′‐GUC CCA AUU CUU AUU CAG GUA GUA GAU‐3′
NM_019371.1	PHD3_3	912	5′‐GAA CAU AAC CUG UUC CAU UUC CUG GAU‐3′

### Real‐time PCR

RNA was isolated using an Aurum Total RNA Mini kit (BioRad, Mississauga, ON). cDNA was synthesized using an iScript cDNA synthesis kit (BioRad). Real‐time PCR was performed using SsoFast EvaGreen real‐time PCR supermix (BioRad) (see Table 2 for primer sequences).

**Table 2:** Real‐time PCR primer sequences. Primer sequences for the following genes

Gene	Sequence
ARNT/HIF1*β*	For: AGC GGT TTG CCA GGT CGG ATG
	Rev: GAT GTG TT GCC AGT TCC CCT CAA
HIF1*α*	For: TCG GAC AGC CTC ACC AGA CAG A
	Rev: TGC TGC TTG AAA AAG GGA GCC A
GLUT2	For: CCT GGC CGG GAT GAT TGG CA
	Rev: AGG CCC GAG GAA GTC CGC AA
Glucokinase	For: GTG TGG AGC CCA GTT GTT GAC T
	Rev: TCG GGG ATG GAG TAC ATC TGG TG
PHD1	For: AGC CGC AGG CCC TGA ATC AA
	Rev: ACG GTC GCT TGC TGG GTG AA
PHD2	For: ACG GCC GAA CGA AAG CCA TG
	Rev: TGC TCG CTC GTC TGC ATC GA
PHD3	For: TCC GCT GGG CGC ATT CGT TTA
	Rev: ACA CGA GGC AAT CCT GGG CTG A
PKM1	For: TGG AGG CCA GCG ATG GAA TCA
	Rev: GCT CTT CAA ACA GCA GGC GGT G

### Statistical analysis

All results are given as mean ± SEM. Statistical significance was assessed by Student's *t* test or by one‐way or two‐way ANOVA followed by multiple comparisons with a Holm–Sidak correction.

## Results

### The PHD inhibitor, EDHB, reduces *β*‐cell glucose‐stimulated insulin secretion

**Figure 1:**
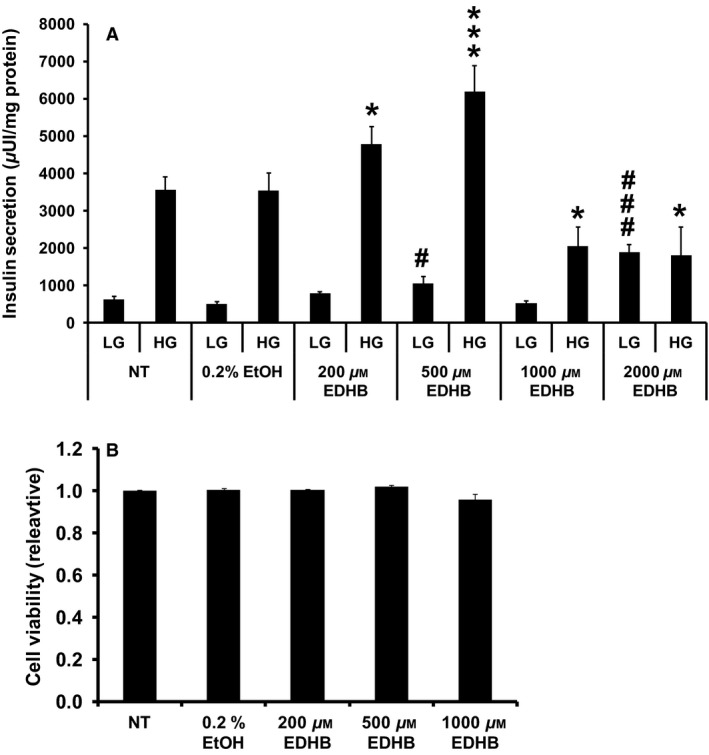
EDHB inhibits GSIS from 832/13 cells. (A) Effects of EDHB on GSIS from 832/13 cells (*n* = 8–12). (B) Relative cell viability. Results represent mean ± SEM. LG, low glucose (2 mmol/L); HG, high glucose (10 mmol/L). #*P *< 0.05, ###*P *< 0.001 for LG NT versus LG EDHB. *P < 0.05, ***P < 0.001 for HG NT versus HG EDHB. 0.2% ethanol was chosen as a control as it was the maximal amount of solvent used at the highest dose of EDHB. NT, no treatment.

To examine the role of PHDs in regulating *β*‐cell insulin secretion, we employed a commonly used PHD inhibitor, EDHB. Adding EDHB during the insulin secretion assay (a 2‐h treatment period with low glucose or high glucose plus or minus drug) statistically increased basal insulin secretion at 500 and 2000 μmol/L, and increased GSIS at 200 and 500 μmol/L in 832/13 cells, whereas 1000 and 2000 μmol/L EDHB led to a reduction in GSIS (Fig. 1A). Treatment with EDHB (200–1000 μmol/L) for 2 h did not affect cell viability (Fig. 1B). Taken together, EDHB effects on GSIS appears to be dependent on the treatment concentration in the clonal 832/13 cells.

### EDHB treatment reduces glucose utilization and alters mitochondrial function

**Figure 2:**
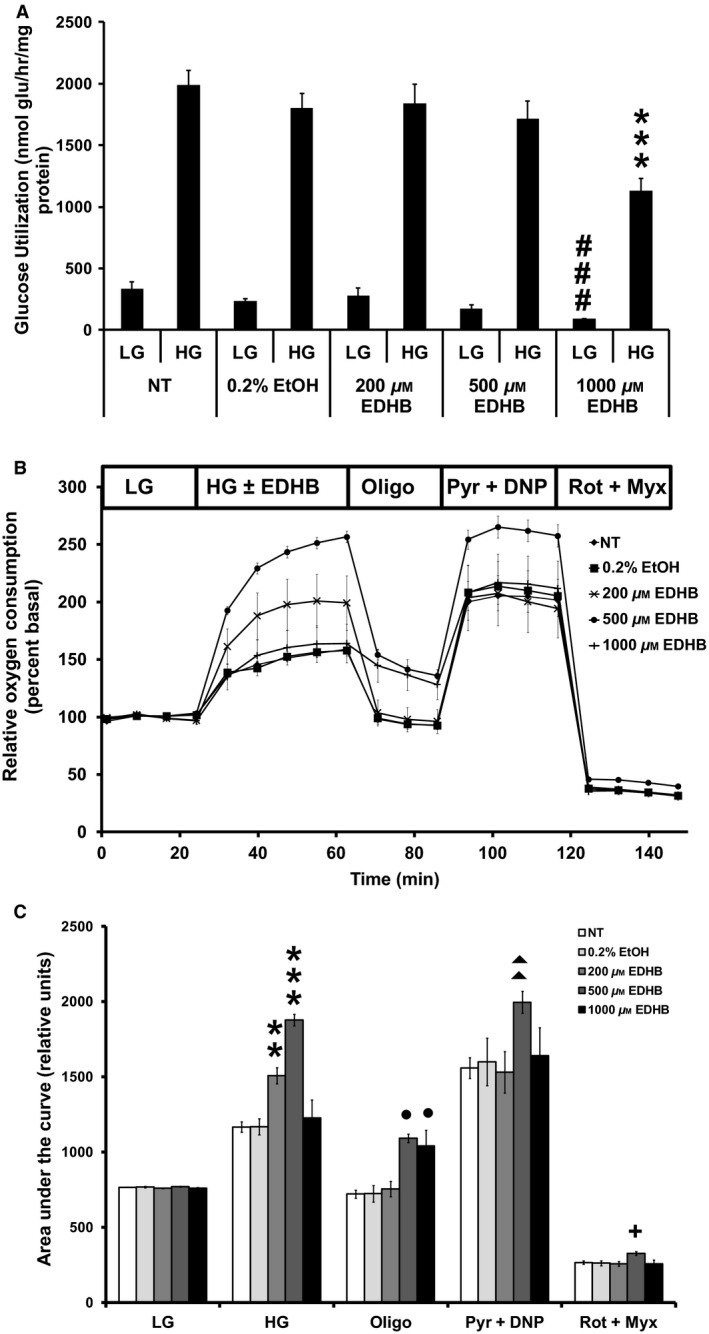
EDHB inhibits glucose utilization, enhances mitochondrial respiration, and uncoupling in 832/13 cells. (A) Glucose utilization (*n* = 15–17), (B) relative oxygen consumption (*n* = 25–27), and (C) AUC of (B). Low (2 mmol/L) or high (10 mmol/L) glucose concentration. ###*P *< 0.001 for LG NT versus LG EDHB. **P < 0.01, ***P < 0.001 for HG NT versus HG EDHB; ●*P *< 0.05 for Oligo NT versus EDHB; ▲▲*P *< 0.01 for Pyr + DNP NT versus EDHB; +*P *< 0.05 for Rot + Myx NT versus EDHB.

Since EDHB treatment affected insulin secretion, we next examined glucose utilization. High glucose induced a 6.0 ± 0.4‐fold increase in glucose utilization in control cells (Fig. 2A). EDHB at 200 and 500 μmol/L had no effect on glucose utilization, whereas 1000 μmol/L EDHB resulted in a significant decrease in glycolytic flux at low glucose and a 43 ± 5% decrease in glycolytic flux at high glucose (Fig. 2A).

We next sought to evaluate the effects of EDHB on mitochondrial function. Consistent with a stimulatory effect on insulin secretion, both 200 and 500 μmol/L EDHB elevated the glucose‐induced oxygen consumption in 832/13 cells (Fig. 2B and C). At 1000 μmol/L EDHB, oxygen consumption levels were normalized to control values. Mitochondrial inhibitors were used to determine overall mitochondrial function. Proton leak, assessed as the difference in respiration between cells treated with oligomycin and rotenone + myxothiazol, was significantly increased in cells treated with 500 μmol/L (104.3 ± 3.1 pmol O_2_/min; *P* < 0.001) and 1000 μmol/L (106.5 ± 14.0 pmol O_2_/min; *P* < 0.01) EDHB as compared to 0.2% ethanol control (61.3 ± 3.0 pmol O_2_/min). Maximal respiration was assessed using the mitochondrial substrate pyruvate and the uncoupler dinitrophenol (DNP). EDHB (500 μmol/L) significantly increased maximal respiration (Fig. 2B and C). These results suggests that part of the reason for increased insulin secretion seen with 200 and 500 μmol/L EDHB was due to enhanced respiration, whereas this was not the case for 1000 μmol/L EDHB.

### EDHB treatment reduces the ATP/ADP ratio and TCA cycle intermediates

**Figure 3:**
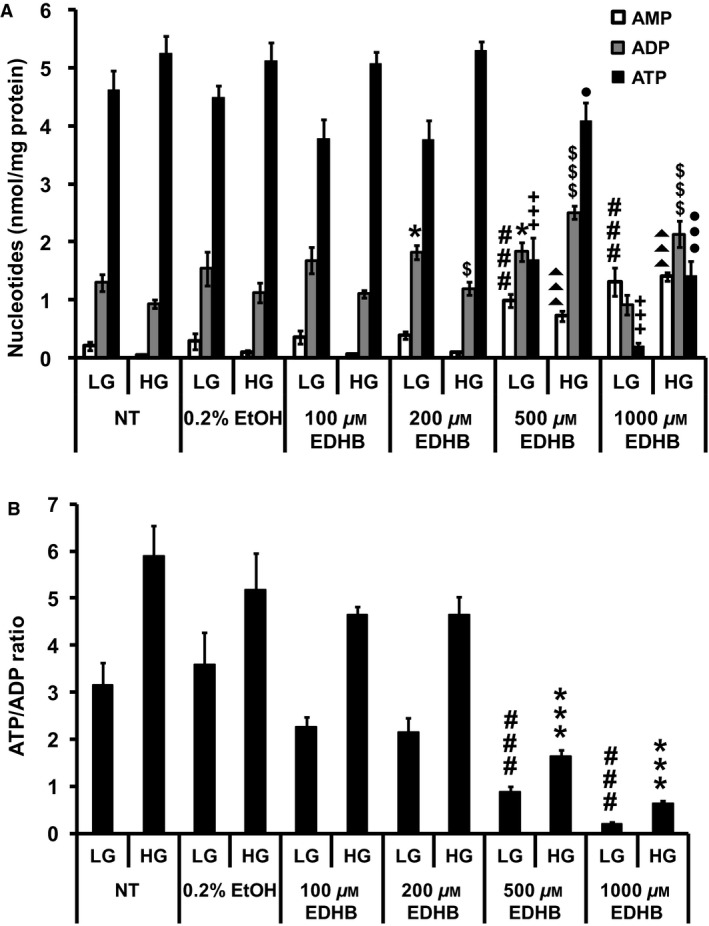
EDHB alters adenine nucleotide levels in 832/13 cells. (A) ATP, ADP, and AMP levels. ###*P *< 0.001 for AMP levels of LG‐treated NT cells versus LG‐treated EDHB cells. *P < 0.05 for ADP levels of LG‐treated NT cells versus LG‐treated EDHB cells. +++*P *< 0.001 for ATP levels of LG‐treated NT cells versus LG‐treated EDHB cells. ▲▲▲*P *< 0.001 for AMP levels of HG‐treated NT cells versus HG‐treated EDHB cells. $*P *< 0.05 and $$$*P *< 0.001 for ADP levels of HG‐treated NT cells versus HG‐treated EDHB cells. ●*P *< 0.05 and ●●●*P *< 0.001 for ATP levels of HG‐treated NT cells versus HG‐treated EDHB cells. (B) ATP:ADP ratio (*n* = 10). LG, low glucose (2 mmol/L); HG, high glucose (10 mmol/L). ###*P *< 0.001 for ATP:ADP ratio of LG‐treated NT cells versus LG‐treated EDHB cells. ***P < 0.001 for ATP/ADP ratio of HG‐treated NT cells versus HG‐treated EDHB cells.

**Figure 4:**
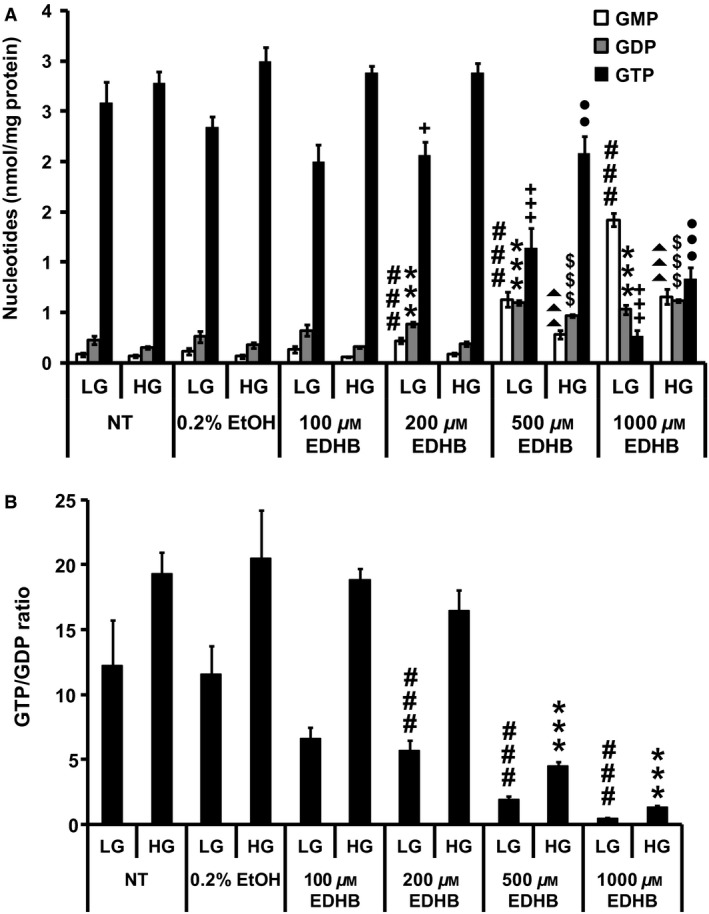
Effects of EDHB on GMP, GDP, and GTP levels in 832/13 cells. (A) GTP, GDP, and GMP levels. ###*P *< 0.001 for GMP levels of LG‐treated NT cells versus LG‐treated EDHB cells. ***P < 0.001 for GDP levels of LG‐treated NT cells versus LG‐treated EDHB cells. +*P *< 0.05 and +++*P *< 0.001 for GTP levels of LG‐treated NT cells versus LG‐treated EDHB cells. ▲▲▲*P *< 0.001 for GMP levels of HG‐treated NT cells versus HG‐treated EDHB cells. $$$*P *< 0.001 for GDP levels of HG‐treated NT cells versus HG‐treated EDHB cells. ●●*P *< 0.01 and ●●●*P *< 0.001 for GTP levels of HG‐treated NT cells versus HG‐treated EDHB cells. (B) GTP/GDP ratio (*n* = 10). NT, no treatment. LG, low glucose (2 mmol/L); HG, high glucose (10 mmol/L). ###*P *< 0.001 for GTP/GDP ratio of LG‐treated NT cells versus LG‐treated EDHB cells. ***P < 0.001 for GTP/GDP ratio of HG‐treated NT cells versus HG‐treated EDHB cells.

Since mitochondrial respiration and uncoupling were affected by EDHB treatment, we next determined nucleotide levels. Consistent with an increase in mitochondrial uncoupling, ATP levels (Fig. 3A) and the ATP:ADP ratio (Fig. 3B) were significantly decreased at 500 and 1000 μmol/L EDHB. Similar results were observed for GTP (Fig. 4A) and the GTP:GDP ratio (Fig. 4B).

**Figure 5:**
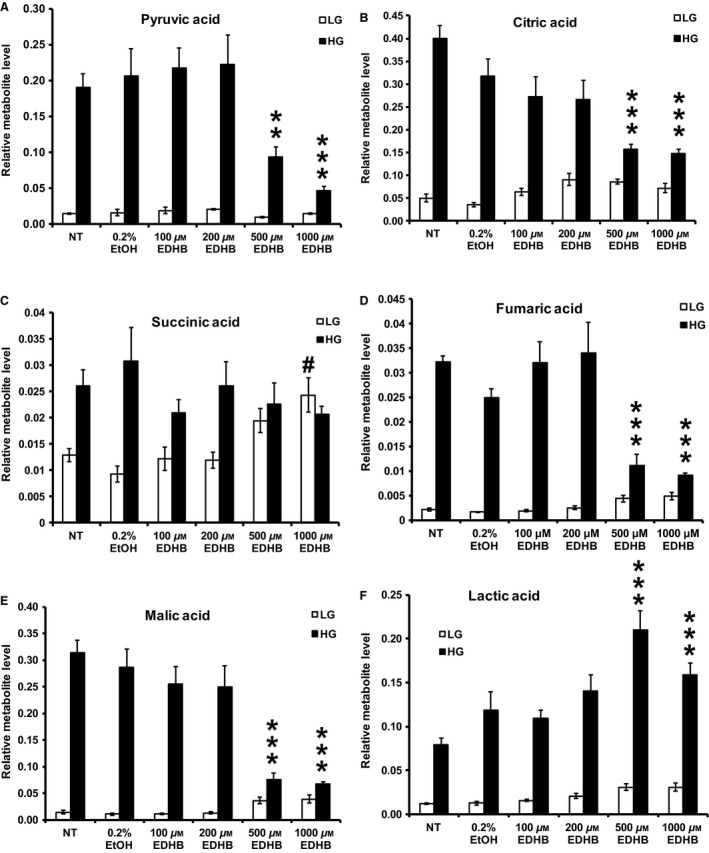
Effects of EDHB on (A) pyruvic acid, (B) citric acid, (C) succinic acid, (D) fumaric acid, (E) malic acid, and (F) lactic acid levels in 832/13 cells. LG, low glucose (2 mmol/L); HG, high glucose (10 mmol/L). #*P *< 0.05 LG‐treated NT cells versus LG‐treated EDHB cells. **P < 0.01, and ***P < 0.001 HG‐treated NT cells versus HG‐treated EDHB cells (*n* = 6).

Tricarboxylic acid cycle intermediates pyruvate (Fig. 5A), citrate (Fig. 5B), fumarate (Fig. 5D), and malate (Fig. 5E) levels were all significantly decreased by 500 and 1000 μmol/L EDHB. No significant effect of EDHB treatment was observed for succinate levels at high glucose, however, low glucose stimulation revealed greater succinate levels at 1000 μmol/L EDHB (Fig. 5C). Lactate levels were significantly elevated at 500 and 1000 μmol/L EDHB (Fig. 5F). Taken altogether, 500 μmol/L EDHB treatment significantly augmented uncoupling, reduced both ATP:ADP ratio, and anaplerosis despite an observed increase in GSIS suggesting a nonmitochondrial mechanism underlying the upregulation in insulin secretion in response to 500 μmol/L EDHB. Meanwhile, treatment of cells with 1000 μmol/L EDHB resulted in no effect on respiration but an increase in mitochondrial uncoupling and inhibition of the glycolysis, ATP/ADP ratio, anaplerosis, and GSIS.

### PHD1 and PHD3 regulate insulin release

**Figure 6:**
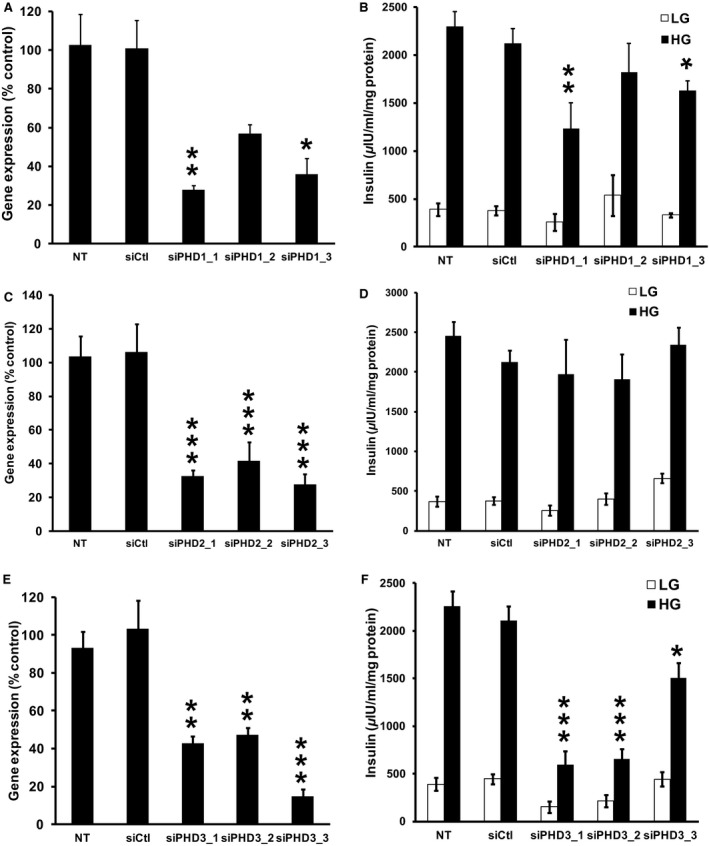
Effects of siRNA‐mediated knockdown of PHD1, PHD2, and PHD3 in 832/13 cells. (A) Effects of three siRNA duplexes targeted against PHD1 (PHD1_1, PHD1_2, PHD1_3) on PHD1 gene expression. (B) The effects of the PHD1 siRNA duplexes on GSIS (*n* = 8–10). (C) Effects of three siRNA duplexes targeted against PHD2 (PHD2_1, PHD2_2, PHD2_3) on PHD2 gene expression. (D) The effects of the PHD2 siRNA duplexes on GSIS (*n* = 8–10). (E) Effects of three siRNA duplexes targeted against PHD3 (PHD3_1, PHD3_2, PHD3_3) on PHD3 gene expression. (F) The effects of the PHD3 siRNA duplexes on GSIS (*n* = 8–10). PHD gene expression was corrected for by an internal control gene (cyclophilin) and then expressed as percent of control, *P < 0.05, **P < 0.01, ***P < 0.001 for NT cells versus siRNA‐treated cells. White and black bars represent secretion at 2 and 10 mmol/L glucose, respectively. siCtrl is a siRNA duplex with no known target. *P < 0.05, **P < 0.01, ***P < 0.001 for 10 mmol/L glucose‐treated NT cells versus 10 mmol/L glucose siRNA‐treated cells.

Consistent with previously published data (Fallon and MacDonald 2008), we found that all three PHD isoforms reside within *β*‐cells (data not shown). Subsequently, we assessed the role of each of these PHDs using three independent siRNA sequences for each isoform. The siRNAs targeting PHD1 reduced gene expression by 75 ± 2%, 46 ± 4%, and 65 ± 8% for siPHD1_1, siPHD1_2, and siPHD1_3, respectively (Fig. 6A), however, only siPHD1_1 and siPHD1_3 were able to significantly suppress GSIS by 47 ± 8% and 29 ± 3%, respectively (Fig. 6B). siRNAs designed against PHD2 lowered gene expression by 62–76% (Fig. 6C), however, none of these targets had a significant effect on insulin secretion (Fig. 6D). Finally, siRNA‐mediated knockdown of PHD3 led to a reduction in PHD3 expression in the range of 51–87% (Fig. 6E) and had the greatest impact on reducing GSIS by 74 ± 5%, 71 ± 3%, and 33 ± 4% for siPHD3_1, siPHD3_2, and siPHD3_3, respectively (Fig. 6F). Altogether, while PHD1 knockdown may have a small effect on GSIS, PHD3 seems to play an essential role in nutrient‐stimulated insulin exocytosis.

### Gene expression

**Figure 7:**
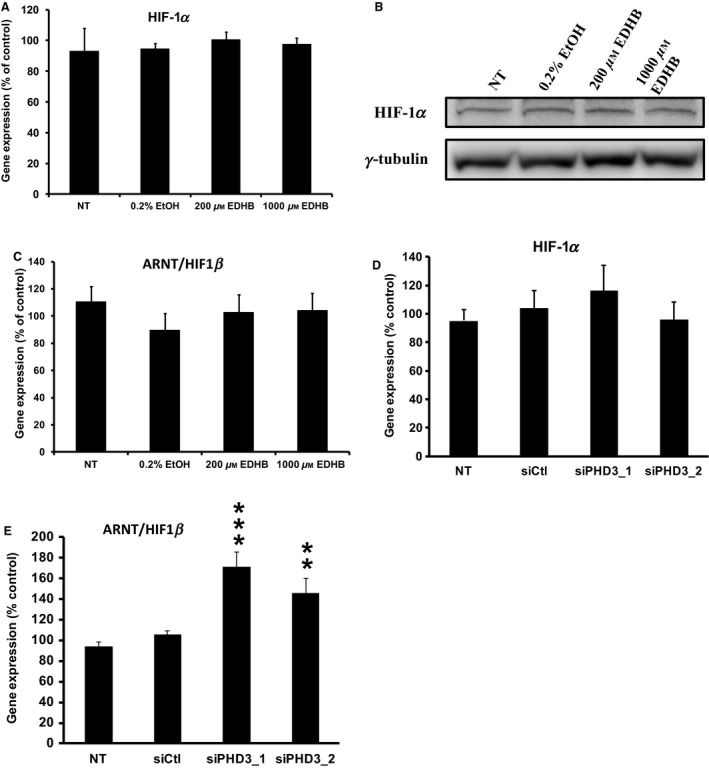
Effects of EDHB and siRNA‐mediated knockdown of PHD3 on gene and protein expression in 832/13 cells. HIF1*α* (A) mRNA expression and (B) protein expression in response to EDHB. mRNA expression of ARNT/HIF1*β* in cells treated with (C) EDHB and (E) a siRNA against PHD3. HIF1*α* mRNA expression (D) in cells treated with a siRNA against PHD3 (*n* = 6–8). Gene expression was corrected for by an internal control gene (cyclophilin) and then expressed as percent of control. *γ* tubulin served as a protein loading control.**P < 0.01, ***P < 0.001.

**Figure 8:**
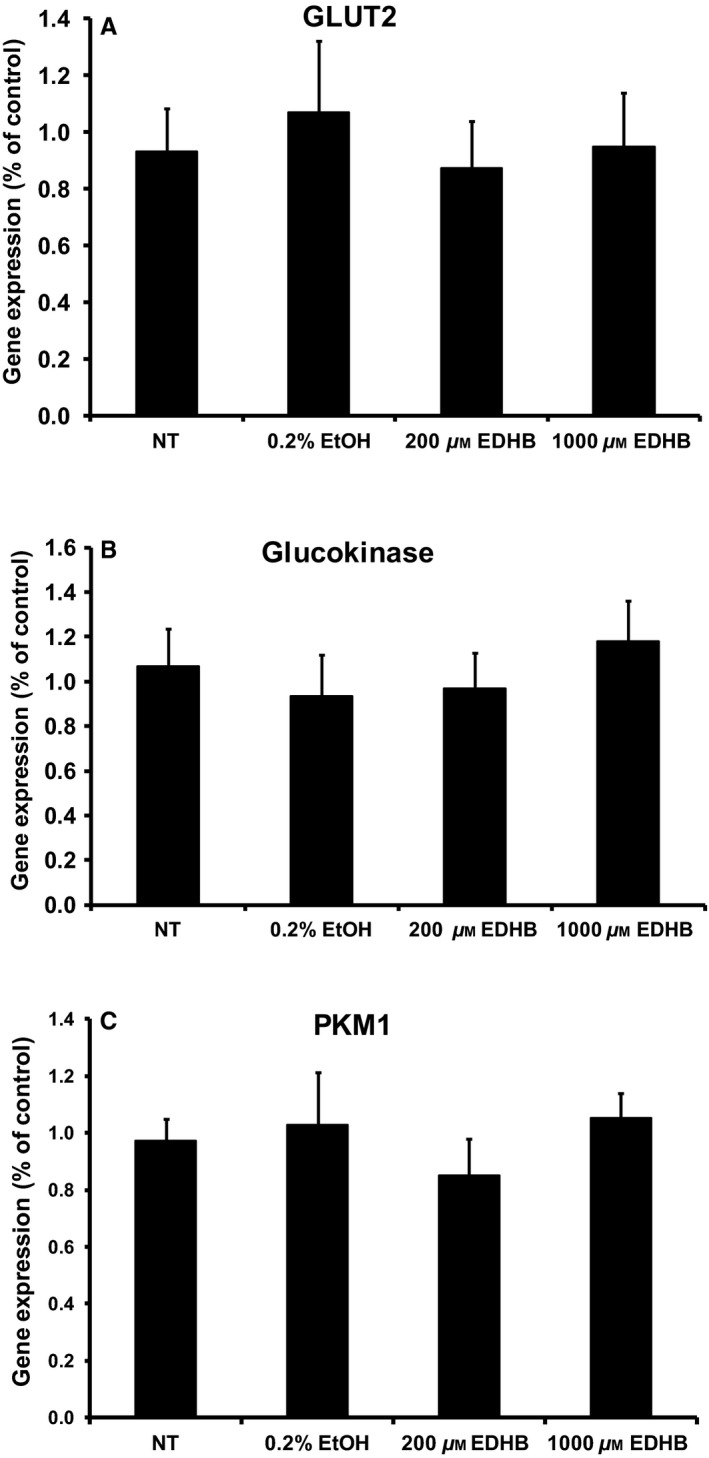
Effects of EDHB on gene expression in 832/13 cells. mRNA expression of (A) glucose transporter 2 (Glut2), (B) glucokinase, (C) pyruvate kinase muscle isozyme 1 (PKM1) (*n* = 6–8). Gene expression was corrected for by an internal control gene (cyclophilin) and then expressed as percent of control.

Since one of the roles of PHDs is to regulate the stability of the hypoxia‐inducible transcription factors, we examined if EDHB altered the expression HIF1*α*, ARNT/HIF1*β*, and key enzymes involved in *β*‐cell glycolysis. A 2‐h EDHB treatment did not affect the mRNA or protein expression of HIF1*α* (Fig. 7A and B) or mRNA expression ARNT/HIF1*β* (Fig. 7C) in 832/13 cells. Consistent with this result, the siRNA targeting of PHD3 in 832/13 cells also did not affect HIF1*α* expression, however, it did significantly elevate ARNT/HIF1*β* mRNA level (Figs. 7D and E, Fig. 8). EDHB also did not affect the expression of glucose transport 2 (GLUT2), glucokinase, or pyruvate kinase muscle isozyme 1 (PKM1) (Fig. 8). These results suggest that the primary target for PHD3 may not be HIF1*α*.

**Figure 9:**
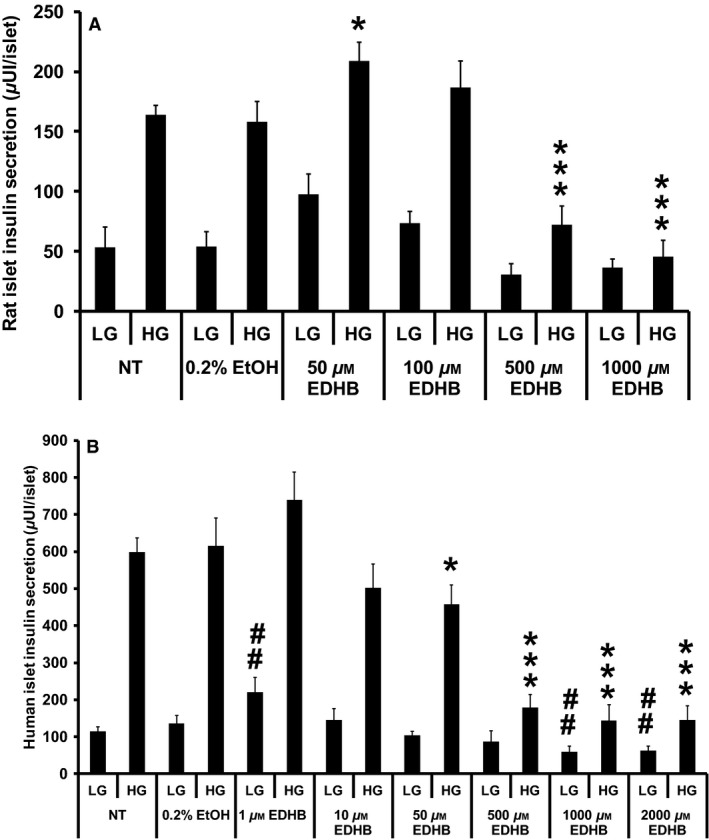
Effects of EDHB on GSIS from rat and human islets. (A) Glucose‐stimulated insulin secretion in response to EDHB from rat islets (*n* = 8–10). (B) Glucose‐stimulated insulin secretion in response to EDHB from human islets (*n* = 15). ##*P *< 0.01 for NT islets versus EDHB‐treated islets at 2 mmol/L glucose. *P < 0.05, and ***P < 0.001 for NT islets versus EDHB‐treated islets at 10 mmol/L glucose.

### EDHB inhibits GSIS from primary rat and human islets

Finally, we examined whether EDHB could inhibit GSIS from isolated rat (Fig. 9A) and human (Fig. 9B) islets. Consistent with the clonal *β*‐cell studies, 50 μmol/L EDHB significantly increased insulin secretion, however, at 500 and 1000 μmol/L EDHB significantly reduced GSIS by 56 ± 9% and 72 ± 8%, respectively, from rat islets (Fig. 9A). No statistical difference was observed with low glucose‐EDHB treatment of rat islets (Fig. 9A). Human islets treated with EDHB also revealed a dose‐dependent decrease in GSIS from 16 to 76% at 50–2000 μmol/L EDHB, respectively (Fig. 9B). Contrary to the rat islets, human islets treated with 1 μmol/L EDHB at low glucose concentrations had a small increase in insulin secretion (Fig. 9B). Taken together, PHD inhibition using pharmacological or siRNA‐mediated knockdown reduces GSIS from clonal *β*‐cells and isolated islets.

## Discussion

In the present study, we demonstrated an essential role of PHD3 in *β*‐cell insulin secretion through pharmacological and genetic approaches. While low doses of EDHB increased GSIS in cells and rat islets, higher doses had a dramatic inhibitory effect on secretion in cultured *β*‐cells and primary islets. At a high EDHB concentrations, blunted GSIS was attributed to reductions in glucose utilization, greater mitochondrial uncoupling, lower ATP:ADP ratios, and a significant reduction in TCA cycle intermediates. We also evaluated the role of the PHD proteins in GSIS through siRNA‐mediated knockdown assays. Altogether we established that siPHD3‐mediated knockdown in *β*‐cells was sufficient to disrupt insulin secretion, suggesting PHD3 is required for normal *β*‐cell function, whereas PHD1 and PHD2 are not. These conclusions are supported by the cytosolic localization of PHD3 which is critical if it is to regulate insulin secretion.

### PHD inhibition alters *β*‐cell metabolic profile

The oxygen‐sensing PHD proteins regulate the stability of HIF1*α* as well as other proline‐containing proteins by catalyzing the hydroxylation of proline residues. This reaction is dependent on the presence of adequate levels of oxygen, iron, and *α*‐KG. *α*‐KG may play a key role as an amplifying signaling molecule regulating insulin release (Odegaard et al. 2010; Huypens et al. 2012). We recently examined the role of anaplerosis in GSIS in an ARNT/HIF1*β* knockdown model (Pillai et al. 2011). We demonstrated that ARNT/HF1*β* siRNA‐mediated knockdown in clonal 832/13 cells resulted in reduced GSIS independent of the ATP:ADP ratio, yet anaplerotic metabolites were dramatically reduced (Pillai et al. 2011), suggesting an important role for ARNT/HIF1*β* in regulating the control of TCA cycle intermediates. In the present study, PHD inhibition with EDHB also led to a significant reduction in the levels TCA intermediates such as citrate, pyruvate, malate, and fumarate, however, this altered metabolic profile was associated with a strong decrease in the ATP:ADP ratio. This distinction between the two studies could be attributed to the proposed mechanistic function of PHDs and ARNT/HIF1*β*. Particularly, ARNT/HIF1*β* is constitutively expressed and under normoxic condition can homodimerize to regulate the gene expression of genes involved in the canonical insulin signaling pathway such as IRS2 and Akt (Gunton et al. 2005), indirectly influencing glycolysis. However, under hypoxic conditions as modeled by EDHB treatment, PHD inhibition prevents HIF1*α* degradation (Warnecke et al. 2003), allowing for HIF1*α* and ARNT/HIF1*β* to directly upregulate glycolytic genes and deterring oxidative phosphorylation (i.e., ATP production) by inducing pyruvate dehydrogenase kinase (PDK) and lactate dehydrogenase (LDH) expression (Fedele et al. 2002). PDK reduces pyruvate entry into the mitochondrial TCA cycle through the inhibition of pyruvate dehydrogenase (PDH), while LDH shunts pyruvate toward lactate formation. As shown here, we observed a significant decrease in pyruvate as well as an elevation in lactate levels upon EDHB treatment. Supporting this concept, it was recently found that PHD3 directly regulates PDH activity (Kikuchi et al. 2014). It is also interesting that EDHB increased GSIS at 500 μmol/L even though ATP, GTP, and anaplerosis inhibited. It is likely that part of the reason for this increased insulin secretion was associated with the dramatic increase in respiration and ATP turnover at 500 μmol/L.

### Targeted PHD knockdown

While all three PHD isoforms are highly expressed in islets (this study and Fallon and MacDonald 2008), we show for the first time that PHD3 has the greatest impact of the three PHDs in regulating insulin exocytosis. PHD3 has not been extensively studied in islets. Whole‐body PHD3 knockout mice develop normally (Takeda et al. 2006), and do not display any discernible phenotype (Minamishima et al. 2009). On the other hand, liver‐specific PHD3 abrogation resulted in increased insulin sensitivity due to an upregulation of insulin signaling associated with greater HIF2*α* stability (Taniguchi et al. 2013), suggesting important tissue‐specific roles. Furthermore, the administration of a pan‐PHD inhibitor (FG‐4497) to wild‐type mice was also shown to be protective against high‐fat‐diet‐induced glucose intolerance (Rahtu‐Korpela et al. 2014). While this finding contradict the current results in *β*‐cells as well as those obtained from VHL KO mice, which display HIF1*α* protein stabilization, delayed glucose tolerance, and blunted GSIS in isolated islets (Cantley et al. 2009; Puri et al. 2009), again this supports the notion of a tissue‐specific function. Alternatively, in addition to its regulation of HIF1*α* and HIF2*α*, PHD inhibition may also affect other pathways linked to GSIS. On that note, NF‐kB suppression leads to impaired GSIS in *β*‐cells and altered whole‐body glucose homeostasis (Norlin et al. 2005). Moreover, Cummins et al. demonstrated that PHD inhibition resulted in NF‐*κ*B activation through the direct hydroxylation of I‐*κ*B kinase *β* in HeLa cells (Cummins et al. 2006). While this has not yet been worked out in islets, further studies are required to elucidate the direct effect of PHD deficiency in islets.

## Conclusion

Recent knockout animal studies have suggested an essential role of PHDs in glucose homeostasis (Matsuura et al. 2013; Taniguchi et al. 2013). In the present study, we determined that *β*‐cell PHD inhibition, through iron‐sequestering resulted in a blunted ATP:ADP ratio, reduced TCA metabolite levels and altered GSIS. Moreover, we provided a putative mechanism for the role of PHDs, as PHD3 siRNA‐mediated knockdown severely affects glucose‐stimulated insulin secretion. Taken together, PHD3 may represent an attractive target for the prevention and treatment of type 2 diabetes.

## Conflict of interest

None declared.
